# [μ-2,3,5,6-Tetra­kis(2-pyrid­yl)pyrazine-κ^6^ *N* ^6^,*N* ^1^,*N* ^2^:*N* ^3^,*N* ^4^,*N* ^5^]bis­[diaqua(dihydrogen *m*-phenylene­diphospho­nato-κ*O*)nickel(II)] dihydrate

**DOI:** 10.1107/S1600536810041279

**Published:** 2010-10-23

**Authors:** Paul DeBurgomaster, Jon Zubieta

**Affiliations:** a Department of Chemistry, Syracuse University, Syracuse, New York 13244, USA

## Abstract

The title compound [Ni_2_(C_6_H_6_O_6_P_2_)_2_(C_24_H_16_N_6_)(H_2_O)_4_]·2H_2_O or [Ni_2_(tpyprz)(1,3-HO_3_PC_6_H_4_PO_3_H)_2_(H_2_O)_4_]·2H_2_O [tpyprz = tetra­kis­(2-pyrid­yl)pyrazine, C_24_H_16_N_6_] is a binuclear complex with a crystallographic inversion center located at the center of the pyrazine ring. The equivalent nickel(II) sites exhibit a distorted {NiO_3_N_3_} octa­hedral coordination, with the three nitro­gen donors of each terminus of the tpyprz ligand in a meridional orientation. An aqua ligand occupies the position *trans* to the pyrazine nitro­gen donor, while the second aqua ligand is *trans* to the oxygen donor of the dihydrogen-1,3-phenyl­diphospho­nate ligand. The Ni—O and Ni—N bond lengths fall in the range 2.011 (3) to 2.089 (3) Å. The protonation sites on the organo­phospho­nate ligand are evident in the significantly longer P—O bonds compared to the unprotonated sites. In the crystal structure, the complex mol­ecules and the solvent water mol­ecules are linked into a three-dimensional hydrogen-bonded framework through O—H⋯O inter­actions between the aqua ligands, the protonated organo­phospho­nate oxygen atoms and the water mol­ecules of crystallization. Intra­molecular π-stacking between the phenyl group of the phospho­nate ligand and a pyridyl group of the tpyprz ligand, at a distance of 3.244 (5) Å between ring centroids, is also observed.

## Related literature

For general background to metal-organo­phospho­nates, see: Alberti *et al.* (1978 ▶); Clearfield (1998 ▶); Finn *et al.* (2003 ▶); Vermeulen (1997 ▶). For nickel–organo­phospho­nates, see: Bauer *et al.* (2008 ▶). For nickel–tetra­kis­(2-pyrid­yl)pyrazine complexes, see: Burkholder *et al.* (2003 ▶); Burkholder & Zubieta (2004 ▶, 2005 ▶). For the use of tetra­kis­(2-pyrid­yl)pyrazine as a component in the construction of metal–organo­phospho­nate materials, see: Armatas *et al.* (2008 ▶). 
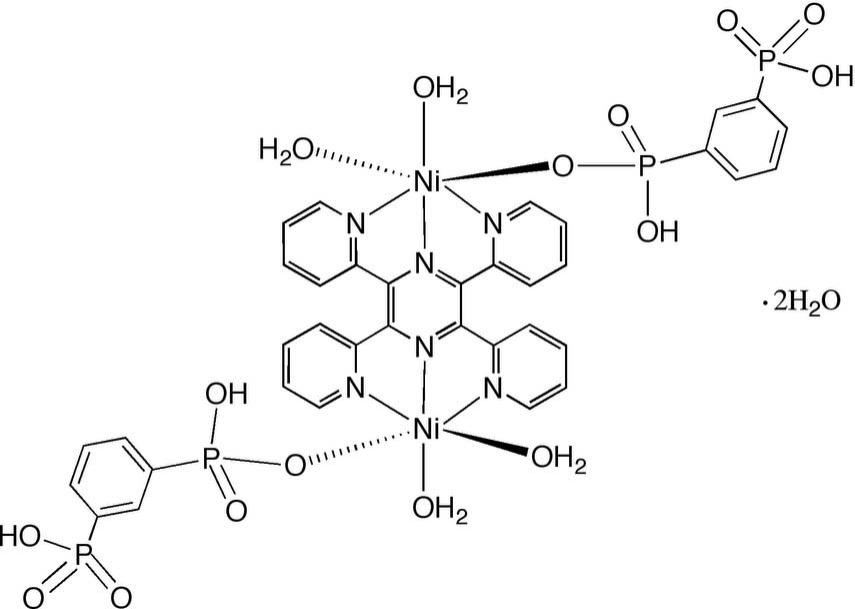


## Experimental

### 

#### Crystal data

[Ni_2_(C_6_H_6_O_6_P_2_)_2_(C_24_H_16_N_6_)(H_2_O)_4_]·2H_2_O
*M* *_r_* = 1086.04
Triclinic, 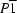

*a* = 7.9702 (6) Å
*b* = 10.0785 (8) Å
*c* = 14.0960 (12) Å
α = 85.386 (2)°
β = 81.707 (1)°
γ = 69.364 (1)°
*V* = 1048.03 (15) Å^3^
*Z* = 1
Mo *K*α radiation
μ = 1.14 mm^−1^
*T* = 298 K
0.20 × 0.14 × 0.11 mm

#### Data collection

Bruker APEX CCD area-detector diffractometer
Absorption correction: multi-scan (*SADABS*; Bruker, 1998 ▶) *T*_min_ = 0.804, *T*_max_ = 0.885
10484 measured reflections
5044 independent reflections
4821 reflections with *I* > 2σ(*I*)
*R*_int_ = 0.023

#### Refinement

*R*[*F* ^2^ > 2σ(*F* ^2^)] = 0.066
*wR*(*F* ^2^) = 0.133
*S* = 1.32
5044 reflections
304 parameters
H-atom parameters constrained
Δρ_max_ = 0.91 e Å^−3^
Δρ_min_ = −0.80 e Å^−3^

### 

Data collection: *SMART* (Bruker, 1998 ▶); cell refinement: *SAINT* (Bruker, 1998 ▶); data reduction: *SAINT*; program(s) used to solve structure: *SHELXS97* (Sheldrick, 2008 ▶); program(s) used to refine structure: *SHELXL97* (Sheldrick, 2008 ▶); molecular graphics: *DIAMOND* (Brandenburg & Putz, 1999 ▶); software used to prepare material for publication: *SHELXTL* (Sheldrick, 2008 ▶).

## Supplementary Material

Crystal structure: contains datablocks I, New_Global_Publ_Block. DOI: 10.1107/S1600536810041279/pk2273sup1.cif

Structure factors: contains datablocks I. DOI: 10.1107/S1600536810041279/pk2273Isup2.hkl

Additional supplementary materials:  crystallographic information; 3D view; checkCIF report

## Figures and Tables

**Table 1:** Hydrogen-bond geometry (Å, °)

*D*—H⋯*A*	*D*—H	H⋯*A*	*D*⋯*A*	*D*—H⋯*A*
O2—H2′⋯O3^i^	0.82	1.91	2.536 (4)	132
O5—H5′⋯O6^ii^	0.82	1.82	2.606 (4)	162
O40—H40*A*⋯O3^iii^	0.84	1.95	2.784 (4)	170
O40—H40*B*⋯O4^iv^	0.88	1.83	2.711 (4)	175
O41—H41*B*⋯O6^iv^	0.83	1.82	2.625 (4)	163
O90—H90*B*⋯O4^v^	0.92	1.84	2.747 (4)	166
O41—H41*A*⋯O90	0.88	1.83	2.643 (4)	151
O90—H90*A*⋯O1	0.92	1.92	2.780 (4)	154

Symmetry codes: (i) 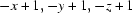; (ii) 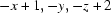; (iii) 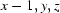; (iv) 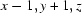; (v) 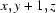.

**Fig. 1.**
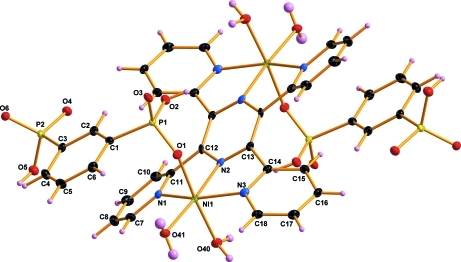
An ellipsoid plot of the structure of the binuclear complex [Ni2(tpyprz)(HO3PC6H4PO3H)2(H2O)4], showing the atom labeling scheme for the asymmetric unit and displacement ellipsoids at the 50% probability level for all non-H atoms. Hydrogen atms are shown as small arbitrary spheres. Color scheme: Ni, green; P, yellow; oxygen, red; nitrogen, blue; carbon, black.

**Fig. 2.**
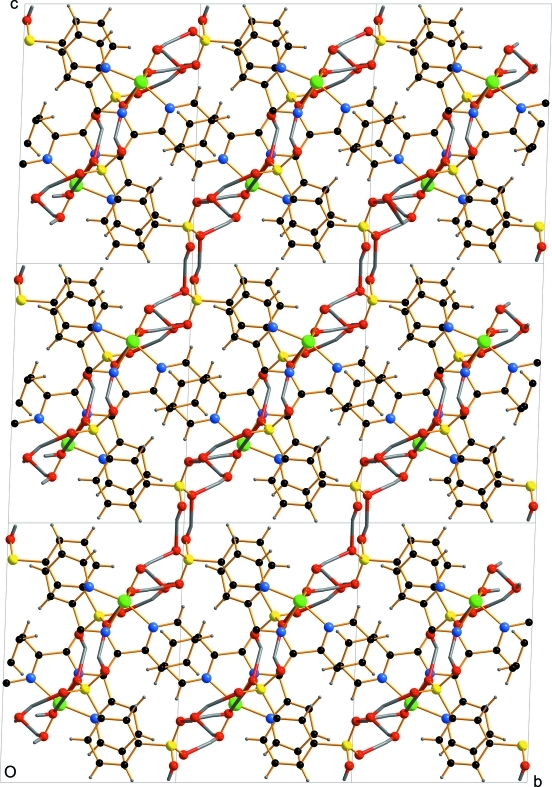
Packing diagram in the bc plane. The hydrogen bonds are shown as rendered multi-band cylinders in red and gray.

## supplementary crystallographic information

### Comment

The chemistry of metal-organophosphonates has witnessed dramatic growth (Clearfield, 1998; Finn *et al.*, 2003; Vermeulen, 1997) since the first reports in the 1970s of the layered metal-organophosphonates (Alberti *et al*., 1978). In our investigations of metal oxide materials, we have used organodiphosphonates as tethers between metal or metal oxide nodes (Armatas *et al.*, 2008). Structural expansion and diversity could be accomplished by introducing additional components, most commonly a *M*(II)-organonitrogen ligand complex. A particularly useful nitrogen donor ligand for structural manipulation is the dipodal tetrakis(2-pyridyl)pyrazine (tpyprz) (Armatas *et al.*, 2008; Bauer *et al.*, 2008). While Cu(II) has generally served as the secondary metal in the *M*(II)-organonitrogen ligand complex, Ni(II)-containing subunits have also been exploited as subunits (Burkholder *et al.*, 2003; Burkholder and Zubieta, 2004; Burkholder and Zubieta, 2005). While the secondary metal *M*(II) bonds to the tpyprz ligand and aqua ligands and/or cluster oxide groups in such materials, the title complex was prepared in the absence of metal oxide, affording the binuclear [Ni_2_(tpyprz)(1,3-HO_3_PC_6_H_4_PO_3_H) (H_2_O)_4_]dihydrate.

As shown in Fig. 1, the structure of the title compound is binuclear, with a crystallographic inversion center at the mid-point of the pyrazine group. The distorted {NiO_3_N_3_} octahedral geometry at the Ni(II) site is defined by the nitrogen donors of the tpyprz ligand in a meridional orientation, two aqua ligands and an oxygen donor from the pendant monodentate 1,3-phenyldiphosphonate ligand. One aqua ligand is *trans* to the pyrazine nitrogen donor of the tpyprz ligand, while the second occupies a position *trans* to the phosphonate oxygen donor. The shortest Ni—N distance is to the pyrazine nitrogen, Ni—N2 of 2.011 (3) Å, while the Ni-pyridyl bond distances are 2.076 (3) Å and 2.089 (3) Å. The Ni—*O*(aqua) distances are 2.015 (3) Å and 2.081 (3) Å, while the Ni—*O*(phosphonate) distance is 2.082 (3) Å.

Charge compensation considerations require that the phenyldiphosphonate ligand be in the doubly deprotonated state [H_2_(O_3_PC_6_H_4_PO_3_)]^2-^. The protonation sites were revealed in the difference Fourier map by peaks adjacent to O2 and O5 at distances consistent with bound hydrogen. The P—O bond lengths support these protonation sites with P—O2 and P—O5 of 1.567 (3) Å and 1.574 (3) Å, respectively, compared to an average P—O distance of 1.515 (4)Å for the remaining P—O distances.

The structure is stabilized by intermolecular hydrogen bonding between the aqua ligands, the P—OH groups and the waters of crystallization. The binuclear complexes and the water of crystallization are linked into a three-dimensional framework through this hydrogen bonding (Fig. 2). There is also intramolecular π-stacking between the phosphonate phenyl ring and a pyridyl group of the tpyprz ligand with a distance of 3.244 (5)Å between centroids. Intermolecular π-stacking between the phosphonate phenyl group and a pyridyl ring of a tpyprz ligand of an adjacent molecule exhibits a distance of 3.584 (5)Å between centroids.

### Experimental

A solution of Ni(CH_3_CO_2_)_2_&bull;4H_2_O (0.074 g, 0.297 mmol), tpyprz (0.085 g, 0.219 mmol) and 1,3-phenyldiphosphonic acid (0.071 g, 0.301 mmol) in water (10 ml) was placed in a Parr acid digestion bomb and heated to 170°C for 48 h. Yellow blocks of the compound suitable for *x*-ray diffraction studies were isolated in 65% yield. Anal Calcd. for C_36_H_40_N_6_Ni_2_O_18_P_4_: C, 39.8; H, 3.68; N. 7.73. Found: C, 39.6; H, 3.75; N, 7.65.

### Refinement

Pyridyl hydrogen atoms were discernable in the difference Fourier map. These hydrogen atoms were placed in calculated positions with C—H = 0.95 Å and included in the riding model approximation with *U*_iso_(H) = 1.2*U*_eq_(C). The hydrogen atoms associated with the oxygen of the phosphonate ligand, the aqua ligands and the water of crystallization were also found on the difference Fourier map. The P—OH hydrogen atoms were included in calculated positions with O—H = 0.82 Å and included in the riding model approximation with *U*_iso_(H) = 1.5*U*_eq_(O). The H atoms of the water molecules were included using the coordinate riding approximation with *U*_iso_(H) free to vary.

### Crystal data

**Table:**

[Ni_2_(C_6_H_6_O_6_P_2_)_2_(C_24_H_16_N_6_)(H_2_O)_4_]·2H_2_O	*Z* = 1
*M**_r_* = 1086.04	*F*(000) = 558
Triclinic, *P*1	*D*_x_ = 1.721 Mg m^−^^3^*D*_m_ = 1.724 (2) Mg m^−^^3^*D*_m_ measured by flotation
Hall symbol: -P 1	Mo *K*α radiation, λ = 0.71073 Å
*a* = 7.9702 (6) Å	Cell parameters from 3874 reflections
*b* = 10.0785 (8) Å	θ = 2.8–28.2°
*c* = 14.0960 (12) Å	µ = 1.14 mm^−^^1^
α = 85.386 (2)°	*T* = 298 K
β = 81.707 (1)°	Block, yellow
γ = 69.364 (1)°	0.20 × 0.14 × 0.11 mm
*V* = 1048.03 (15) Å^3^	

### Data collection

**Table:**

Bruker APEX CCD area-detector diffractometer	5044 independent reflections
Radiation source: fine-focus sealed tube	4821 reflections with *I* > 2σ(*I*)
graphite	*R*_int_ = 0.023
φ and ω scans	θ_max_ = 28.1°, θ_min_ = 2.8°
Absorption correction: multi-scan (*SADABS*; Bruker, 1998)	*h* = −10→10
*T*_min_ = 0.804, *T*_max_ = 0.885	*k* = −13→13
10484 measured reflections	*l* = −18→18

### Refinement

**Table:**

Refinement on *F*^2^	Primary atom site location: structure-invariant direct methods
Least-squares matrix: full	Secondary atom site location: difference Fourier map
*R*[*F*^2^ > 2σ(*F*^2^)] = 0.066	Hydrogen site location: inferred from neighbouring sites
*wR*(*F*^2^) = 0.133	H-atom parameters constrained
*S* = 1.32	*w* = 1/[σ^2^(*F*_o_^2^) + (0.0262*P*)^2^ + 4.5072*P*] where *P* = (*F*_o_^2^ + 2*F*_c_^2^)/3
5044 reflections	(Δ/σ)_max_ < 0.001
304 parameters	Δρ_max_ = 0.91 e Å^−^^3^
0 restraints	Δρ_min_ = −0.80 e Å^−^^3^

### Special details

**Table:**

Geometry. All e.s.d.'s (except the e.s.d. in the dihedral angle between two l.s. planes) are estimated using the full covariance matrix. The cell e.s.d.'s are taken into account individually in the estimation of e.s.d.'s in distances, angles and torsion angles; correlations between e.s.d.'s in cell parameters are only used when they are defined by crystal symmetry. An approximate (isotropic) treatment of cell e.s.d.'s is used for estimating e.s.d.'s involving l.s. planes.
Refinement. Refinement of *F*^2^ against ALL reflections. The weighted *R*-factor *wR* and goodness of fit *S* are based on *F*^2^, conventional *R*-factors *R* are based on *F*, with *F* set to zero for negative *F*^2^. The threshold expression of *F*^2^ > σ(*F*^2^) is used only for calculating *R*-factors(gt) *etc*. and is not relevant to the choice of reflections for refinement. *R*-factors based on *F*^2^ are statistically about twice as large as those based on *F*, and *R*- factors based on ALL data will be even larger.

### Fractional atomic coordinates and isotropic or equivalent isotropic displacement parameters (Å^2^)

**Table:**

	*x*	*y*	*z*	*U*_iso_*/*U*_eq_	
Ni1	−0.07580 (6)	0.67510 (5)	0.69829 (3)	0.00726 (13)	
P1	0.36680 (13)	0.53302 (10)	0.64046 (7)	0.00822 (19)	
P2	0.44896 (13)	0.03282 (10)	0.85848 (7)	0.0097 (2)	
O1	0.1916 (4)	0.6581 (3)	0.6542 (2)	0.0117 (5)	
O2	0.3592 (4)	0.4229 (3)	0.5703 (2)	0.0121 (5)	
H2'	0.3391	0.4617	0.5177	0.018*	
O3	0.5296 (4)	0.5787 (3)	0.60760 (19)	0.0118 (5)	
O4	0.4279 (4)	−0.0163 (3)	0.76433 (19)	0.0125 (5)	
O5	0.2948 (4)	0.0145 (3)	0.9359 (2)	0.0151 (6)	
H5'	0.3043	0.0409	0.9878	0.023*	
O6	0.6315 (4)	−0.0397 (3)	0.8936 (2)	0.0131 (6)	
O40	−0.3505 (4)	0.7120 (3)	0.7354 (2)	0.0135 (6)	
H40A	−0.3999	0.6770	0.6998	0.020 (13)*	
H40B	−0.4191	0.8019	0.7417	0.019 (13)*	
O41	−0.0740 (4)	0.7601 (3)	0.82264 (19)	0.0125 (6)	
H41A	0.0129	0.7965	0.8162	0.041 (17)*	
H41B	−0.1766	0.8105	0.8461	0.036 (17)*	
O90	0.1372 (4)	0.9039 (3)	0.7492 (2)	0.0190 (6)	
H90A	0.1811	0.8321	0.7059	0.038 (17)*	
H90B	0.2363	0.9222	0.7635	0.049 (19)*	
N1	−0.0352 (4)	0.4705 (3)	0.7536 (2)	0.0101 (6)	
N2	−0.0652 (4)	0.5726 (3)	0.5797 (2)	0.0089 (6)	
N3	−0.1409 (4)	0.8436 (3)	0.5978 (2)	0.0107 (6)	
C1	0.3907 (5)	0.4355 (4)	0.7530 (3)	0.0102 (7)	
C2	0.4116 (5)	0.2912 (4)	0.7604 (3)	0.0114 (7)	
H2	0.4237	0.2423	0.7052	0.014*	
C3	0.4145 (5)	0.2200 (4)	0.8494 (3)	0.0102 (7)	
C4	0.3956 (6)	0.2949 (4)	0.9323 (3)	0.0145 (8)	
H4	0.3961	0.2488	0.9922	0.017*	
C5	0.3764 (6)	0.4373 (4)	0.9252 (3)	0.0149 (8)	
H5	0.3654	0.4861	0.9803	0.018*	
C6	0.3734 (5)	0.5074 (4)	0.8364 (3)	0.0132 (8)	
H6	0.3598	0.6031	0.8324	0.016*	
C7	−0.0568 (5)	0.4352 (4)	0.8473 (3)	0.0138 (8)	
H7A	−0.0539	0.4973	0.8919	0.017*	
C8	−0.0831 (6)	0.3106 (4)	0.8799 (3)	0.0175 (8)	
H8A	−0.0939	0.2877	0.9452	0.021*	
C9	−0.0931 (6)	0.2202 (4)	0.8141 (3)	0.0177 (8)	
H9	−0.1152	0.1371	0.8347	0.021*	
C10	−0.0699 (5)	0.2546 (4)	0.7170 (3)	0.0137 (8)	
H10	−0.0761	0.1950	0.6716	0.016*	
C11	−0.0373 (5)	0.3793 (4)	0.6889 (3)	0.0113 (7)	
C12	−0.0117 (5)	0.4313 (4)	0.5878 (3)	0.0080 (7)	
C13	−0.0605 (5)	0.6454 (4)	0.4965 (3)	0.0087 (7)	
C14	−0.1448 (5)	0.8029 (4)	0.5091 (3)	0.0100 (7)	
C15	−0.2340 (5)	0.8978 (4)	0.4408 (3)	0.0124 (7)	
H15	−0.2421	0.8667	0.3818	0.015*	
C16	−0.3113 (5)	1.0407 (4)	0.4623 (3)	0.0134 (8)	
H16	−0.3729	1.1066	0.4180	0.016*	
C17	−0.2956 (5)	1.0839 (4)	0.5507 (3)	0.0146 (8)	
H17	−0.3395	1.1796	0.5649	0.017*	
C18	−0.2134 (5)	0.9818 (4)	0.6170 (3)	0.0132 (8)	
H18	−0.2083	1.0101	0.6774	0.016*	

### Atomic displacement parameters (Å^2^)

**Table:**

	*U*^11^	*U*^22^	*U*^33^	*U*^12^	*U*^13^	*U*^23^
Ni1	0.0073 (2)	0.0063 (2)	0.0088 (2)	−0.00260 (17)	−0.00161 (17)	−0.00129 (17)
P1	0.0087 (4)	0.0077 (4)	0.0083 (4)	−0.0029 (3)	−0.0011 (3)	−0.0003 (3)
P2	0.0117 (5)	0.0082 (4)	0.0089 (4)	−0.0027 (4)	−0.0022 (4)	0.0002 (3)
O1	0.0107 (13)	0.0096 (13)	0.0151 (13)	−0.0043 (10)	−0.0013 (10)	0.0003 (10)
O2	0.0142 (13)	0.0122 (13)	0.0115 (13)	−0.0064 (11)	−0.0020 (10)	−0.0004 (10)
O3	0.0134 (13)	0.0162 (14)	0.0085 (12)	−0.0082 (11)	−0.0019 (10)	−0.0014 (10)
O4	0.0164 (14)	0.0101 (13)	0.0112 (13)	−0.0041 (11)	−0.0022 (11)	−0.0032 (10)
O5	0.0158 (14)	0.0177 (14)	0.0125 (14)	−0.0071 (12)	−0.0009 (11)	−0.0006 (11)
O6	0.0126 (13)	0.0123 (13)	0.0119 (13)	−0.0009 (11)	−0.0022 (10)	−0.0003 (10)
O40	0.0120 (13)	0.0124 (14)	0.0174 (14)	−0.0050 (11)	−0.0018 (11)	−0.0050 (11)
O41	0.0116 (13)	0.0121 (13)	0.0140 (13)	−0.0041 (11)	−0.0005 (11)	−0.0042 (11)
O90	0.0168 (15)	0.0183 (15)	0.0248 (16)	−0.0097 (12)	0.0010 (12)	−0.0063 (13)
N1	0.0075 (14)	0.0113 (15)	0.0109 (15)	−0.0017 (12)	−0.0027 (12)	−0.0003 (12)
N2	0.0062 (14)	0.0081 (15)	0.0136 (15)	−0.0026 (12)	−0.0040 (12)	−0.0017 (12)
N3	0.0091 (15)	0.0101 (15)	0.0142 (16)	−0.0046 (12)	−0.0015 (12)	−0.0023 (12)
C1	0.0090 (17)	0.0116 (18)	0.0100 (17)	−0.0039 (14)	−0.0001 (14)	−0.0004 (14)
C2	0.0106 (17)	0.0122 (18)	0.0126 (18)	−0.0041 (14)	−0.0040 (14)	−0.0022 (14)
C3	0.0095 (17)	0.0091 (17)	0.0128 (18)	−0.0035 (14)	−0.0036 (14)	0.0008 (14)
C4	0.019 (2)	0.0152 (19)	0.0095 (17)	−0.0062 (16)	−0.0027 (15)	0.0001 (15)
C5	0.020 (2)	0.0149 (19)	0.0112 (18)	−0.0061 (16)	−0.0019 (15)	−0.0052 (15)
C6	0.0157 (19)	0.0077 (17)	0.0169 (19)	−0.0046 (15)	−0.0031 (15)	−0.0006 (14)
C7	0.0126 (18)	0.0165 (19)	0.0117 (18)	−0.0037 (15)	−0.0004 (14)	−0.0041 (15)
C8	0.022 (2)	0.015 (2)	0.0118 (18)	−0.0031 (16)	−0.0012 (16)	0.0041 (15)
C9	0.021 (2)	0.0093 (18)	0.020 (2)	−0.0053 (16)	0.0039 (17)	0.0028 (15)
C10	0.0171 (19)	0.0086 (17)	0.0157 (19)	−0.0052 (15)	0.0001 (15)	−0.0021 (14)
C11	0.0096 (17)	0.0092 (17)	0.0145 (18)	−0.0015 (14)	−0.0024 (14)	−0.0031 (14)
C12	0.0095 (16)	0.0093 (17)	0.0076 (16)	−0.0052 (13)	−0.0054 (13)	0.0025 (13)
C13	0.0083 (16)	0.0064 (16)	0.0128 (17)	−0.0030 (13)	−0.0040 (14)	−0.0010 (13)
C14	0.0091 (17)	0.0085 (17)	0.0132 (18)	−0.0043 (14)	−0.0004 (14)	−0.0008 (14)
C15	0.0141 (18)	0.0119 (18)	0.0112 (18)	−0.0044 (15)	−0.0009 (14)	−0.0017 (14)
C16	0.0108 (17)	0.0112 (18)	0.0151 (19)	−0.0006 (14)	−0.0013 (15)	0.0028 (15)
C17	0.0165 (19)	0.0083 (18)	0.018 (2)	−0.0044 (15)	0.0008 (16)	−0.0030 (15)
C18	0.0115 (18)	0.0140 (19)	0.0154 (19)	−0.0063 (15)	0.0012 (15)	−0.0040 (15)

### Geometric parameters (Å, °)

**Table:**

Ni1—N2	2.011 (3)	C1—C2	1.402 (5)
Ni1—O41	2.016 (3)	C2—C3	1.393 (5)
Ni1—N1	2.076 (3)	C2—H2	0.9300
Ni1—O40	2.082 (3)	C3—C4	1.403 (5)
Ni1—O1	2.082 (3)	C4—C5	1.385 (6)
Ni1—N3	2.089 (3)	C4—H4	0.9300
P1—O1	1.516 (3)	C5—C6	1.385 (6)
P1—O3	1.525 (3)	C5—H5	0.9300
P1—O2	1.566 (3)	C6—H6	0.9300
P1—C1	1.795 (4)	C7—C8	1.377 (6)
P2—O4	1.504 (3)	C7—H7A	0.9300
P2—O6	1.515 (3)	C8—C9	1.380 (6)
P2—O5	1.574 (3)	C8—H8A	0.9300
P2—C3	1.805 (4)	C9—C10	1.387 (6)
O2—H2'	0.8200	C9—H9	0.9300
O5—H5'	0.8200	C10—C11	1.388 (5)
O40—H40A	0.8445	C10—H10	0.9300
O40—H40B	0.8824	C11—C12	1.489 (5)
O41—H41A	0.8831	C12—C13^i^	1.406 (5)
O41—H41B	0.8318	C13—C12^i^	1.406 (5)
O90—H90A	0.9213	C13—C14	1.504 (5)
O90—H90B	0.9235	C14—C15	1.386 (5)
N1—C7	1.342 (5)	C15—C16	1.392 (5)
N1—C11	1.353 (5)	C15—H15	0.9300
N2—C13	1.336 (5)	C16—C17	1.388 (6)
N2—C12	1.336 (5)	C16—H16	0.9300
N3—C18	1.339 (5)	C17—C18	1.382 (6)
N3—C14	1.355 (5)	C17—H17	0.9300
C1—C6	1.394 (5)	C18—H18	0.9300
			
N2—Ni1—O41	174.66 (12)	C3—C2—H2	119.6
N2—Ni1—N1	78.70 (13)	C1—C2—H2	119.6
O41—Ni1—N1	96.31 (12)	C2—C3—C4	119.1 (4)
N2—Ni1—O40	92.64 (12)	C2—C3—P2	120.8 (3)
O41—Ni1—O40	88.91 (11)	C4—C3—P2	120.1 (3)
N1—Ni1—O40	86.27 (12)	C5—C4—C3	120.2 (4)
N2—Ni1—O1	87.69 (12)	C5—C4—H4	119.9
O41—Ni1—O1	91.29 (11)	C3—C4—H4	119.9
N1—Ni1—O1	99.42 (11)	C4—C5—C6	120.4 (4)
O40—Ni1—O1	174.25 (11)	C4—C5—H5	119.8
N2—Ni1—N3	78.95 (13)	C6—C5—H5	119.8
O41—Ni1—N3	106.20 (12)	C5—C6—C1	120.5 (4)
N1—Ni1—N3	156.87 (13)	C5—C6—H6	119.7
O40—Ni1—N3	88.87 (12)	C1—C6—H6	119.7
O1—Ni1—N3	85.55 (12)	N1—C7—C8	122.5 (4)
O1—P1—O3	112.44 (16)	N1—C7—H7A	118.7
O1—P1—O2	112.43 (16)	C8—C7—H7A	118.7
O3—P1—O2	110.23 (15)	C7—C8—C9	119.0 (4)
O1—P1—C1	107.08 (17)	C7—C8—H8A	120.5
O3—P1—C1	110.95 (16)	C9—C8—H8A	120.5
O2—P1—C1	103.31 (17)	C8—C9—C10	119.2 (4)
O4—P2—O6	115.53 (16)	C8—C9—H9	120.4
O4—P2—O5	108.24 (16)	C10—C9—H9	120.4
O6—P2—O5	110.12 (16)	C9—C10—C11	118.9 (4)
O4—P2—C3	109.86 (17)	C9—C10—H10	120.6
O6—P2—C3	106.30 (17)	C11—C10—H10	120.6
O5—P2—C3	106.41 (17)	N1—C11—C10	121.7 (4)
P1—O1—Ni1	133.25 (16)	N1—C11—C12	113.1 (3)
P1—O2—H2'	109.5	C10—C11—C12	125.1 (3)
P2—O5—H5'	109.5	N2—C12—C13^i^	117.7 (3)
Ni1—O40—H40A	116.6	N2—C12—C11	112.5 (3)
Ni1—O40—H40B	115.1	C13^i^—C12—C11	129.9 (3)
H40A—O40—H40B	106.5	N2—C13—C12^i^	118.1 (3)
Ni1—O41—H41A	109.6	N2—C13—C14	112.0 (3)
Ni1—O41—H41B	112.7	C12^i^—C13—C14	129.8 (3)
H41A—O41—H41B	117.7	N3—C14—C15	122.0 (3)
H90A—O90—H90B	106.4	N3—C14—C13	113.7 (3)
C7—N1—C11	118.6 (3)	C15—C14—C13	124.0 (3)
C7—N1—Ni1	124.9 (3)	C14—C15—C16	118.5 (4)
C11—N1—Ni1	113.8 (3)	C14—C15—H15	120.7
C13—N2—C12	124.1 (3)	C16—C15—H15	120.7
C13—N2—Ni1	116.3 (2)	C17—C16—C15	119.4 (4)
C12—N2—Ni1	116.6 (2)	C17—C16—H16	120.3
C18—N3—C14	118.6 (3)	C15—C16—H16	120.3
C18—N3—Ni1	126.3 (3)	C18—C17—C16	118.6 (4)
C14—N3—Ni1	113.3 (2)	C18—C17—H17	120.7
C6—C1—C2	118.9 (3)	C16—C17—H17	120.7
C6—C1—P1	119.3 (3)	N3—C18—C17	122.6 (4)
C2—C1—P1	121.6 (3)	N3—C18—H18	118.7
C3—C2—C1	120.9 (3)	C17—C18—H18	118.7
			
O3—P1—O1—Ni1	−179.15 (19)	O4—P2—C3—C4	168.7 (3)
O2—P1—O1—Ni1	55.7 (3)	O6—P2—C3—C4	−65.6 (3)
C1—P1—O1—Ni1	−57.0 (3)	O5—P2—C3—C4	51.8 (4)
N2—Ni1—O1—P1	−64.4 (2)	C2—C3—C4—C5	−0.7 (6)
O41—Ni1—O1—P1	110.4 (2)	P2—C3—C4—C5	177.4 (3)
N1—Ni1—O1—P1	13.8 (2)	C3—C4—C5—C6	0.8 (6)
N3—Ni1—O1—P1	−143.5 (2)	C4—C5—C6—C1	−0.4 (6)
N2—Ni1—N1—C7	−166.2 (3)	C2—C1—C6—C5	−0.1 (6)
O41—Ni1—N1—C7	15.7 (3)	P1—C1—C6—C5	174.7 (3)
O40—Ni1—N1—C7	−72.8 (3)	C11—N1—C7—C8	−0.8 (6)
O1—Ni1—N1—C7	108.1 (3)	Ni1—N1—C7—C8	159.6 (3)
N3—Ni1—N1—C7	−151.1 (3)	N1—C7—C8—C9	−2.0 (6)
N2—Ni1—N1—C11	−5.1 (3)	C7—C8—C9—C10	2.3 (6)
O41—Ni1—N1—C11	176.9 (3)	C8—C9—C10—C11	0.0 (6)
O40—Ni1—N1—C11	88.4 (3)	C7—N1—C11—C10	3.2 (6)
O1—Ni1—N1—C11	−90.8 (3)	Ni1—N1—C11—C10	−159.2 (3)
N3—Ni1—N1—C11	10.1 (5)	C7—N1—C11—C12	−179.9 (3)
N1—Ni1—N2—C13	−171.4 (3)	Ni1—N1—C11—C12	17.7 (4)
O40—Ni1—N2—C13	103.0 (3)	C9—C10—C11—N1	−2.8 (6)
O1—Ni1—N2—C13	−71.3 (3)	C9—C10—C11—C12	−179.3 (4)
N3—Ni1—N2—C13	14.6 (3)	C13—N2—C12—C13^i^	2.5 (6)
N1—Ni1—N2—C12	−10.2 (3)	Ni1—N2—C12—C13^i^	−157.1 (3)
O40—Ni1—N2—C12	−95.8 (3)	C13—N2—C12—C11	−178.3 (3)
O1—Ni1—N2—C12	89.9 (3)	Ni1—N2—C12—C11	22.1 (4)
N3—Ni1—N2—C12	175.8 (3)	N1—C11—C12—N2	−26.0 (4)
N2—Ni1—N3—C18	165.3 (3)	C10—C11—C12—N2	150.7 (4)
O41—Ni1—N3—C18	−16.1 (3)	N1—C11—C12—C13^i^	153.1 (4)
N1—Ni1—N3—C18	150.2 (3)	C10—C11—C12—C13^i^	−30.2 (6)
O40—Ni1—N3—C18	72.4 (3)	C12—N2—C13—C12^i^	−2.5 (6)
O1—Ni1—N3—C18	−106.2 (3)	Ni1—N2—C13—C12^i^	157.1 (3)
N2—Ni1—N3—C14	0.5 (3)	C12—N2—C13—C14	174.8 (3)
O41—Ni1—N3—C14	179.0 (2)	Ni1—N2—C13—C14	−25.5 (4)
N1—Ni1—N3—C14	−14.6 (5)	C18—N3—C14—C15	−5.4 (5)
O40—Ni1—N3—C14	−92.4 (3)	Ni1—N3—C14—C15	160.7 (3)
O1—Ni1—N3—C14	89.0 (3)	C18—N3—C14—C13	−179.6 (3)
O1—P1—C1—C6	−51.1 (3)	Ni1—N3—C14—C13	−13.5 (4)
O3—P1—C1—C6	72.0 (3)	N2—C13—C14—N3	25.5 (4)
O2—P1—C1—C6	−169.9 (3)	C12^i^—C13—C14—N3	−157.6 (4)
O1—P1—C1—C2	123.6 (3)	N2—C13—C14—C15	−148.6 (4)
O3—P1—C1—C2	−113.4 (3)	C12^i^—C13—C14—C15	28.3 (6)
O2—P1—C1—C2	4.7 (4)	N3—C14—C15—C16	4.3 (6)
C6—C1—C2—C3	0.2 (6)	C13—C14—C15—C16	178.0 (4)
P1—C1—C2—C3	−174.5 (3)	C14—C15—C16—C17	0.5 (6)
C1—C2—C3—C4	0.2 (6)	C15—C16—C17—C18	−4.2 (6)
C1—C2—C3—P2	−177.9 (3)	C14—N3—C18—C17	1.5 (6)
O4—P2—C3—C2	−13.2 (4)	Ni1—N3—C18—C17	−162.6 (3)
O6—P2—C3—C2	112.5 (3)	C16—C17—C18—N3	3.2 (6)
O5—P2—C3—C2	−130.2 (3)		

Symmetry codes: (i) −*x*, −*y*+1, −*z*+1.

### Hydrogen-bond geometry (Å, °)

**Table:**

*D*—H···*A*	*D*—H	H···*A*	*D*···*A*	*D*—H···*A*
O2—H2'···O3^ii^	0.82	1.91	2.536 (4)	132
O5—H5'···O6^iii^	0.82	1.82	2.606 (4)	162
O40—H40A···O3^iv^	0.84	1.95	2.784 (4)	170
O40—H40B···O4^v^	0.88	1.83	2.711 (4)	175
O41—H41B···O6^v^	0.83	1.82	2.625 (4)	163
O90—H90B···O4^vi^	0.92	1.84	2.747 (4)	166
O41—H41A···O90	0.88	1.83	2.643 (4)	151
O90—H90A···O1	0.92	1.92	2.780 (4)	154

Symmetry codes: (ii) −*x*+1, −*y*+1, −*z*+1; (iii) −*x*+1, −*y*, −*z*+2; (iv) *x*−1, *y*, *z*; (v) *x*−1, *y*+1, *z*; (vi) *x*, *y*+1, *z*.
